# Analysis of the SOFA score, quick-SOFA, and SIRS criteria in burn patients with infection

**DOI:** 10.1590/0034-7167-2024-0111

**Published:** 2025-08-08

**Authors:** Francielli Mary Pereira Gimenez, Lucienne Tibery Queiroz Cardoso, Gilselena Kerbauy, Tiemi Matsuo, Cintia Magalhães Carvalho Grion

**Affiliations:** I Universidade Estadual de Londrina. Londrina, Paraná, Brazil

**Keywords:** Burn Units, Sepsis, Screening, Organ Dysfunction Scores, Systemic Inflammatory Response Syndrome., Unidades de Quemados, Sepsis, Cribado, Puntuaciones de Disfunción Orgánica, Síndrome de Respuesta Inflamatoria Sistémica.

## Abstract

**Objectives:**

to evaluate the SOFA score, qSOFA, SIRS criteria, and risk factors for hospital mortality in burn victims with suspected infection admitted to an intensive care unit.

**Methods:**

a retrospective longitudinal study conducted at a public hospital between January 2017 and January 2020. We analyzed sepsis diagnostic scores at two time points: hospital admission and date of infection.

**Results:**

of the 279 patients analyzed, 251 developed an infection. Among these, 145 had a positive SIRS score at the time of the burn, and 112 remained positive at the first documented infection. The SOFA score increased in 187 patients following the burn injury, and 34 remained positive at the time of infection.

**Conclusions:**

the scores on the dates of burn injury and infection did not show variations in SIRS or SOFA compatible with sepsis diagnosis. Age, total body surface area burned, and SOFA score were independent risk factors for mortality.

## RESUMEN

**Objetivos:**

evaluar la puntuación SOFA, qSOFA, los criterios SIRS y los factores de riesgo de mortalidad hospitalaria en víctimas de quemaduras con sospecha de infección ingresadas en unidades de cuidados intensivos.

**Métodos:**

estudio longitudinal retrospectivo realizado en un hospital público entre enero de 2017 y enero de 2020. Se analizaron los puntajes para el diagnóstico de sepsis en dos momentos: admisión hospitalaria y fecha de la infección.

**Resultados:**

de 279 pacientes, 251 desarrollaron infección. Entre ellos, 145 presentaron SIRS positivo en la quemadura, manteniéndose 112 positivos en la infección. La variación del SOFA fue positiva en 187 pacientes en la quemadura, y 34 continuaron positivos en la infección.

**Conclusiones:**

los puntajes en las fechas de la quemadura e infección no mostraron variación de SIRS y SOFA compatible con el diagnóstico de sepsis. La edad, la superficie corporal quemada y el SOFA fueron factores de riesgo independientes para la mortalidad.

## INTRODUCTION

The skin serves a protective function against external agents, including microorganisms and environmental aggressions. When a burn occurs, leading to skin destruction, the patient becomes more susceptible to infections, which are the most frequent and severe complications in burn victims^(1)^.

Burn injuries trigger an intense inflammatory response and alter clinical and laboratory inflammatory markers, mimicking the diagnosis of sepsis^(2)^. When an infection occurs following a burn injury, identifying sepsis can be challenging, raising concerns about the effectiveness of the tools recommended for sepsis screening and diagnosis in these patients.

A life-threatening infection is classified as sepsis. The Third International Consensus Definitions for Sepsis and Septic Shock, known as *Sepsis-3*, defines sepsis as potentially fatal organ dysfunction caused by a dysregulated host response to infection. The diagnosis of sepsis is established when a suspected or confirmed infection is associated with organ dysfunction, identified as a variation of 2 or more points in the Sequential Organ Failure Assessment (SOFA) score^(3)^. Currently, the most commonly used tools for sepsis screening and diagnosis in hospital settings are the Systemic Inflammatory Response Syndrome (SIRS), SOFA, and quick SOFA (qSOFA); however, these criteria are not specifically designed for burn victims.

The first consensus definition of sepsis introduced Systemic Inflammatory Response Syndrome (SIRS) as an inflammatory response to a variety of severe clinical insults^(4)^, emphasizing its crucial role in screening potentially infected patients.

The SOFA score was originally developed to assess dysfunction in six organ systems in patients with suspected sepsis. The qSOFA score was later designed as a simplified version of SOFA, specifically for use in emergency settings, to identify patients at higher risk of prolonged hospitalization, increased mortality, and the need for intensive care^(3,5)^.

The costs associated with treating burn patients are high, estimated at approximately $30,000 per patient^(6)^. Globally, the incidence of sepsis in severely burned patients ranges from 3% to 30%, with pneumonia being the most common infectious focus^(7)^. In Brazil, sepsis is the leading cause of death in these cases^(8)^, and the average monthly operational cost of a burn unit is R$1,277,582.21 (US$220,607)^(9)^.

Given these facts, analyzing the tools commonly used to identify infection and sepsis among hospitalized burn victims is essential. Timely treatment can reduce mortality risk, although early recognition remains a challenge.

## OBJECTIVES

To evaluate the SOFA score, qSOFA, SIRS criteria, and risk factors for hospital mortality in burn victims with suspected infection admitted to an intensive care unit.

## METHODS

### Ethical aspects

The National Research Ethics Commission (CONEP) and the institution’s ethics committee approved this study through the Certificate of Presentation for Ethical Consideration (CAAE). We requested and obtained a waiver of the Informed Consent Form (ICF).

### Study design, period, and setting

This retrospective longitudinal study was conducted in a burn intensive care unit (BICU) between January 2017 and January 2020. We used the STROBE checklist from the EQUATOR Network.

### Population or sample; inclusion and exclusion criteria

The University Hospital of the State University of Londrina (HU-UEL) is a large, public tertiary-care facility with 330 beds. The burn treatment center, located within HU-UEL, serves as a referral unit for burn patients. It consists of ten ward beds, an emergency room, an operating room, and six intensive care unit (BICU) beds. We conducted this study in the BICU. The hospital is classified as a high-complexity facility and functions as a referral center for the Unified Health System (SUS) in northern Paraná, Brazil. It provides specialized care for burn victims from approximately 250 municipalities in Paraná and over 100 cities from other states. The burn treatment center specializes in managing firstto third-degree burn injuries in both adults and children.

The sample consisted of the medical records of all burn patients admitted to the BICU between January 2017 and January 2020, totaling 299 patients. We excluded 20 patients, of whom 14 were under 18 years of age, and 6 had incomplete medical records. A record was considered incomplete if missing data prevented the calculation of the scores or if it lacked variables essential to the study.

### Study protocol

A team of four researchers collected the data after receiving training on the study definitions and the calculation of the applied scores. The team included a monitor responsible for auditing data entry and quality and three collaborators who rotated in daily data collection. The auditing researcher was a nurse with a doctorate in health sciences, while the three collaborating researchers were undergraduate medical students. The demographic and clinical data collected included the date of the burn injury, hospital and BICU admission dates, age, sex, weight, height, Abbreviated Burn Severity Index (ABSI)^(10)^, total body surface area burned (TBSA), burn etiology, causative agent, reason for the burn, and presence of chronic disease.

We considered “hospital admission” as the date when the burn injury event was assessed. The SOFA, qSOFA, and SIRS scores were collected at two time points: on the date of the burn injury and on the date of the first suspected infection episode. Data on the infection date, infection site, and laboratory results for lactate and C-reactive protein (CRP) were also collected. Hospital discharge dates and hospital outcomes were recorded.

The scores were calculated following the original descriptions by their respective authors^(3,4)^. For the SOFA score, we collected the ratio of arterial oxygen pressure to inspired oxygen fraction (PaO₂/FiO₂) and the use of mechanical ventilation, blood platelet count, total bilirubin levels, blood pressure levels and use of vasopressor drugs, Glasgow Coma Scale score, blood creatinine levels, and urine output. For the qSOFA score, respiratory rate, systolic blood pressure, and altered mental status were collected. For SIRS, we collected body temperature, heart rate, respiratory rate, white blood cell count, and the presence of immature white blood cell forms in the blood. We calculated the values for ABSI, SOFA, qSOFA, SIRS, and BMI and recorded lactate and CRP results as documented in the patient’s medical records.

We considered the baseline SOFA score zero when the patient had no preexisting comorbidity that would contribute to the score. A variation of 2 or more points was classified as a positive ∆SOFA, whereas a variation of less than 2 points was considered a negative ∆SOFA^(3)^. We assessed the burn related ∆SOFA at unit admission by comparing the SOFA score on the admission date to the baseline score. The ∆SOFA for infection diagnosis was determined by comparing the SOFA score on the infection date with the score recorded at unit admission.

For each qSOFA score component, one point was assigned according to the following criteria: respiratory rate ≥ 22 breaths/min, systolic blood pressure ≤ 100 mmHg, and altered mental status. A qSOFA score of 2 or 3 points was classified as positive (+)^(3)^.

SIRS was scored based on the following criteria: body temperature > 38°C or < 36°C, heart rate > 90 beats/min, respiratory rate > 20 breaths/min or PaCO₂ < 32 mmHg (< 4.3 kPa), and white blood cell count > 12,000 cells/mm^3^, < 4,000 cells/mm^3^, or > 10% immature forms. SIRS was classified as positive (+) if the patient met two or more of these criteria^(4)^.

Infection occurrence was the primary outcome of the study, with analyses and comparisons focusing on the first infection following the burn injury. The attending physician diagnosed the infection based on the patient’s symptoms and the diagnostic criteria established by the local hospital infection control committee. As a secondary outcome, we assessed the patient’s vital status at hospital discharge.

We defined comorbidity according to the Charlson Comorbidity Index (CCI) criteria^(11,12)^. Finally, body mass index (BMI) was used to identify obesity, its severity levels, and overweight conditions.

### Analysis of results and statistics

For sample size calculation, we considered a 95% two-sided significance level, an 80% detection probability, a 1:1 ratio between patients with positive and negative ∆SOFA, a 70% proportion of patients with negative ∆SOFA and infection, and a 99.9% proportion of patients with positive ∆SOFA and infection. Based on these parameters, the estimated sample size was 62 infected patients. Given that the infection rate in a burn treatment center (BTC) can range from 20% to 65% of admitted patients, we planned a total sample of 250 patients admitted to the BICU, ensuring the observation of at least 62 infection episodes.

In descriptive statistics, continuous variables were presented as mean, standard deviation (SD), median, and interquartile range (IQR). Categorical nominal variables were expressed as absolute and relative frequencies (%). All variables were displayed in tables.

In analytical statistics, we compared categorical variables using Fisher’s exact test. The Mann Whitney nonparametric test was applied for non-normally distributed data and/or variance heterogeneity. The Wilcoxon signed-rank test was used for paired samples to assess individual differences. Categorical variables were analyzed with the chi-square test, while McNemar’s test was employed to evaluate discordances. To assess risk factors for the outcome “death at hospital discharge,” we conducted a proportional hazards analysis using the Cox model, with stepwise selection for variable inclusion. The variables included in the model were age, female sex, total body surface area burned, presence of comorbidities, and the SIRS, qSOFA, and SOFA scores at hospital admission.

## RESULTS

During the study period, 299 patients were admitted to the BICU, with 20 patients excluded, resulting in an analyzed sample of 279 patients between January 2017 and January 2020. Among the studied patients, 182 (65.2%) survived and were discharged, while 97 (34.8%) died. Of the 279 patients analyzed, 186 (66.7%) were male. The mean age of the entire sample was 43.3 years (SD = 17.1). The mean BMI was 25.8 kg/m^2^ (SD = 4.4). The most common chronic conditions were smoking (58; 20.8%), alcohol use (54; 19.4%), drug use (43; 15.4%), and systemic arterial hypertension (38; 13.6%) (Table 1).

**Table 1:** Mean and standard deviation of age and BMI for the total sample, survivors, and deceased patients. Number and percentage by sex and chronic diseases in the total sample, survivors, and deceased patients, Londrina, Paraná, Brazil, January 2017 to January 2020

Characteristics	Total (n = 279)	Survivors (n = 182)	Deceased (n = 97)	*p* value
Age (Mean ± SD)	43.3 ± 17.1	41.0 ± 15.7	47.8 ± 18.7	0.004**^a^**
Sex, n (%) Female Male	93 (33.3) 186 (66.7)	62 (66.7) 120 (64.5)	31 (33.3) 66 (35.5)	0.722^b^
BMI (Mean ± SD)	25.8 ± 4.4	25.7 ± 4.6	25.9 ± 4.0	0.506^a^
Chronic diseases, n (%) Asthma/COPD Cancer Depression Diabetes Mellitus Drug use Epilepsy Schizophrenia Alcohol use Hypertension HIV CHF Smoking	4 (1.4) 1 (0.4) 6 (2.2) 4 (1.4) 43 (15.4) 7 (2.5) 3 (1.1) 54 (19.4) 38 (13.6) 1 (0.4) 7 (2.5) 58 (20.8)	4 (2.2) - 4 (2.2) 3 (1.6) 25 (13.7) 6 (3.3) 2 (1.1) 30 (16.5) 25 (13.7) - 5 (2.7) 37 (20.3)	- 1 (1.0) 2 (2.1) 1 (1.0) 18 (18.6) 1 (1.0) 1 (1.0) 24 (24.7) 13 (13.4) 1 (1.0) 2 (2.1) 21 (21.6)	0.302^c^ 0.348^c^ 1.000^c^ 1.000^c^ 0.288^c^ 0.428^c^ 1.000^c^ 0.096^c^ 0.938^c^ 0.348^c^ 1.000^c^ 0.796^c^

SD - standard deviation; BMI - body mass index; COPD - chronic obstructive pulmonary disease; HIV - human immunodeficiency virus; CHF - congestive heart failure;

a Mann-Whitney;

b Chi-square;

c Fisher’s exact test.

Regarding the time from burn injury to BICU admission, the mean duration was 2.7 days (SD = 3.5), and the mean ABSI score was 6.7 (SD = 2.2). The mean TBSA burned was 25.6% (SD = 17.6). Inhalation injury was present in 55 patients (19.7%). Thermal burns accounted for 83.1% of cases. The most common burn agents were alcohol (104; 37.5%), flames (45; 16.2%), gasoline (33; 11.9%), and hot liquids (32; 11.6%). Domestic accidents were the most frequent cause of burns (58.5%), followed by workplace accidents (25.6%).

Of the 279 patients analyzed, 251 (89.9%) developed one or more episodes of infection during hospitalization. No infections were detected at hospital admission. The mean time from burn injury to the first documented infection was 4.8 days (SD = 4.0). The infection site was undetermined in 39 cases (16%). The most common infection focus was pulmonary (107; 42%), followed by skin and soft tissue infection (45; 18%), bloodstream infection (32; 13%), urinary tract infection (26; 10%), and ear infection (2; 1%).

Regarding laboratory test variations, there was a decrease in mean lactate levels and an increase in mean C-reactive protein (CRP) levels between the burn injury and the first documented infection (Table 2).

**Table 2:** Median and interquartile range of lactate and C-reactive protein at admission and first infection, Londrina, Paraná, Brazil, January 2017 to January 2020

Variables	n	Admission	1^st^ infection	DIF	*p* value
Lactate (Median ± IQR)	250	2.0 ± 1.3	1.9 ± 1.1	-0.1 ± 0.8	0.012^a^
CRP (Median ± IQR)	248	112.5 ± 195.8	197.9 ± 44.8	-14.8 ± (-102.5)	<0.001^a^

DIF - Differential Items Functioning, IQR - interquartile range; CRP - C-reactive protein;

a Wilcoxon.

At the time of the burn injury, SIRS was positive in 153 patients (54.8%) and negative in 126 (45.2%). In the first documented infection, SIRS was positive in 170 patients (67.7%) and negative in 81 (32.3%). Regarding qSOFA, 172 patients (68.5%) had a negative score, and 79 (31.5%) had a positive score at the time of the burn. During the first documented infection, 147 patients (58.6%) had a negative qSOFA score, while 104 (41.4%) had a positive score. For ∆SOFA at the time of the burn injury, 64 patients (25.5%) had a score variation of less than 2 points (negative ∆SOFA), while 187 (74.5%) had a variation of 2 or more points (positive ∆SOFA). At the time of the first documented infection, ∆SOFA was negative in 192 patients (76.5%) and positive in 59 (23.5%) (Table 3).

**Table 3:** Number and percentage of SIRS, qSOFA, and ∆SOFA at admission and first infection, Londrina, Paraná, Brazil, January 2017 to January 2020

Variables	Admission	1^st^ infection
SIRS, n (%)		
Positive	153 (54.8)	170 (67.7)
Negative	126 (45.2)	81 (32.3)
qSOFA, n (%)		
Positive	172 (68.5)	147 (58.6)
Negative	79 (31.5)	104 (41.4)
∆SOFA, n (%)		
< 2	64 (25.5)	192 (76.5)
≥ 2	187 (74.5)	59 (23.5)

*SIRS - Systemic Inflammatory Response Syndrome, qSOFA - quick Sequential Organ Failure Assessment, SOFA - Sequential Organ Failure Assessment.*

*∆SOFA at admission = SOFA admission - SOFA baseline. ∆SOFA at infection = SOFA infection - SOFA admission.*

In the score analysis, 145 patients (57.7%) had a positive SIRS score at the time of the burn injury. In contrast, 112 (44.6%) remained positive, and 33 (13.1%) transitioned to a negative SIRS score at the first documented infection. Regarding qSOFA, 79 patients (31.5%) had a positive score at the time of the burn, of whom 62 (24.7%) remained positive and 17 (6.8%) transitioned to a negative qSOFA score at the first documented infection. For the SOFA score, 187 patients (74.5%) had a positive ∆SOFA at the time of the burn injury, of whom 34 (13.5%) remained positive and 153 (61.0%) transitioned to a negative ∆SOFA at the first documented infection (Table 4).

**Table 4:** Number and percentage of positive SIRS, qSOFA, and ∆SOFA variables at admission and at infection diagnosis, Londrina, Paraná, Brazil, January 2017 to January 2020

Admission	Infection		*p* value
	Positive variable	Negative variable	
SIRS Positive Negative	112 (44.6%) 58 (23.1%)	33 (13.1%) 48 (19.1%)	0.011^a^
qSOFA Positive Negative	62 (24.7%) 42 (16.7%)	17 (6.8%) 130 (51.8%)	0.002^a^
∆SOFA ≥ 2 Positive Negative	34 (13.5%) 25 (10.0%)	153 (61.0%) 39 (15.5%)	<0.001^a^

*SIRS - Systemic Inflammatory Response Syndrome, qSOFA - quick Sequential Organ Failure Assessment, SOFA - Sequential Organ Failure Assessment.*

∆SOFA at admission = SOFA admission - SOFA baseline. ∆SOFA at infection = SOFA infection - SOFA admission.

a McNemar.

A higher mortality rate was observed among patients with infection and a positive ∆SOFA score (49.2%) compared to those with infection and a negative ∆SOFA score (34.9%; p = 0.049). Although the mortality rate among infected patients was high in both groups, it was greater among those with a SOFA variation of 2 or more points.

To analyze risk factors for mortality, we performed a logistic regression using the stepwise method. The independent factors associated with in-hospital mortality showed that each additional year of age increased mortality by 2.6%, each 1% increase in TBSA burned increased the likelihood of death by 3.6%, and each additional point in the SOFA score increased mortality by 12.7% (p < 0.001) (Table 5).

**Table 5:** Hazard ratio and confidence interval for mortality risk factors in the full model and stepwise model, Londrina, Paraná, Brazil, January 2017 to January 2020

Variáveis	Full model Hazard ratio (95% CI)	*p* value	Stepwise model Hazard ratio (95% CI)	*p* value
Age	1.021 (1.008 - 1.034)	<0.001	1.026 (1.014 - 1.039)	< 0.001
Female sex	0.800 (0.511 - 1.253)	0.330		
TBSA burned	1.029 (1.018 - 1.040)	<0.001	1.036 (1.025 - 1.047)	< 0.001
Comorbidity	1.141 (0.690 - 1.886)	0.607		
SIRS at admission	0.579 (0.334 - 1.003)	0.051	0.574 (0.336 - 0.981)	0.043
qSOFA at admission	0.917 (0.636 - 1.320)	0.640		
SOFA at admission	1.107 (1.026 - 1.195)	0.009	1.127 (1.067 - 1.190)	< 0.001

*CI - confidence interval; TBSA - total body surface area burned; SIRS - Systemic Inflammatory Response Syndrome; qSOFA - quick Sequential Organ Failure Assessment; SOFA - Sequential Organ Failure Assessment.*

Among the laboratory tests and prognostic scores evaluated, the area under the ROC curve was calculated, but all curves demonstrated low accuracy in predicting infection or death. The area under the ROC curve for the SOFA score at the time of the burn injury to predict infection was 0.323, for lactate at the time of the burn to predict infection was 0.371, and for CRP at the time of the burn to predict infection was 0.330. The area under the ROC curve for the SOFA score at the time of the burn injury to predict mortality was 0.252, for lactate to predict mortality was 0.337, and for CRP to predict mortality was 0.396.

## DISCUSSION

The results of this study show that more than half of the patients already had altered inflammatory markers and organ dysfunction immediately after the burn injury, even without infection. At the time of infection diagnosis, these markers remained positive in some patients but became negative in others. Among patients with negative markers at the time of the burn, not all later tested positive for sepsis identification at the time of infection. On the other hand, burn patients with infection had a high risk of death, regardless of the positivity of the analyzed markers. These findings suggest that conventional sepsis screening and diagnostic tools perform poorly in burn patients with suspected infection. Age, total body surface area burned, and SOFA score were independent risk factors for in-hospital mortality.

A variation of 2 or more points in the SOFA score had a similar frequency at the time of burn injury and infection among the studied patients. This criterion was adopted for sepsis diagnosis in the Sepsis-3 consensus^(3)^. Another study also found that a SOFA score of 2 or more points did not perform well in identifying sepsis in burn patients^(13)^. The authors suggested that a SOFA score of 6 or more points might be a better criterion for diagnosing sepsis in this population. Our findings indicate that burn patients with infection and a SOFA score variation of less than 2 points may still have a high risk of death, even if they do not meet the Sepsis-3 consensus diagnostic criteria for sepsis. Thus, increasing the SOFA score variation threshold to 6 points would not be appropriate, as it would further reduce the sensitivity of sepsis diagnosis in our patients.

The SOFA score at the time of the burn remained an independent factor associated with mortality, suggesting its potential as a criterion for evaluating these patients. This score has demonstrated greater prognostic accuracy for in-hospital mortality than SIRS and qSOFA criteria in patients admitted to an intensive care unit^(14)^. In addition to SOFA, age and total body surface area burned were also associated with death among burn patients in our study, in line with findings from other authors^(5)^. Advanced age and the destruction of the body’s natural protective barrier increase the risk of infections and other complications, consequently raising morbidity and mortality rates in burn patients^(15)^.

In this study, burn cases were more frequent among men. This finding is consistent with national literature, as epidemiological studies on burn victims have shown a higher incidence of these accidents in males^(16,17)^. The mean age of affected patients is similar to findings in other studies, with a prevalence in the adult population^(18,19)^. This situation has a negative socioeconomic and family impact, as this age group is actively engaged in the labor market and/or household responsibilities, contributing to family income and caregiving^(20)^. Regarding chronic diseases, smoking, alcohol consumption, and drug use were the most prevalent conditions. One study found that 19.37% of burn patients were smokers, 15.03% consumed alcohol, and 6.83% used drugs^(16)^.

In this study, the mean TBSA burned exceeded 20%, similar to results found by other authors^(18)^. Regarding etiology, thermal burns were the most prevalent, and domestic accidents were the most common cause. These findings have also been reported by other authors^(19)^. The mean score on the Abbreviated Burn Severity Index (ABSI) was similar to values reported in other studies^(18,20)^. This score classifies these patients within the “moderately severe life-threatening” category, requiring continuous monitoring and specialized care.

Respiratory complications are a significant concern in burn patients, particularly those with smoke inhalation injuries. However, patients with extensive burns who do not experience smoke inhalation frequently develop pulmonary complications due to immobility and hypoventilation caused by pain. The need for multiple anesthetic procedures can lead to atelectasis and subsequent pneumonia. Sedatives and neuromuscular blockers promotes bronchial secretion retention and tracheal aspiration^(21)^. Other authors have also identified the lungs as the most frequent site of infection^(13,22)^. Compared to the mean TBSA burned, the mean length of hospital stay was consistent with findings from other studies^(18)^.

The median lactate level at the time of infection diagnosis showed a slight decrease compared to the median at the time of the burn, while the median CRP level at infection was higher than at the time of the burn. Thermal injury triggers the recruitment of neutrophils and macrophages. These cells release inflammatory cytokines, which activate CRP production, while inflammation leads to tissue hypoperfusion and lactate formation^(2,23)^. Other authors have reported elevated CRP levels in burn patients even without infection^(24-26)^. Therefore, CRP and lactate should not be used as standalone diagnostic tools for detecting infection in burn patients^(24,25)^. The low accuracy of the ROC curves for SOFA, lactate, and CRP in predicting infection or mortality suggests the need to identify new biomarkers.

Diagnosing infectious complications and identifying signs of sepsis remain significant challenges in the care of critically burned patients. No single variable has sufficient sensitivity and specificity to detect sepsis. However, when used together, these variables can improve diagnostic accuracy^(27)^. The use of early warning tools in nursing practice has a major impact on decision-making in critical situations, improving patient care across various hospital settings. Therefore, these tools can positively influence patient care and enhance bedside clinical reasoning^(28)^.

Despite advances in sepsis prevention, early identification, and treatment, this condition continues to have a high incidence in hospital settings, particularly among burn patients, who are more susceptible to this complication^(29)^. Standardizing the use of scoring systems at patient admission allows for developing protocols that enable multidisciplinary teams to plan, guide, and tailor care to individual patients. This facilitates prompt and high-quality resource allocation, ultimately improving survival rates in this population^(18)^.

Burn injuries are preventable, and prevention efforts can significantly reduce their incidence. An effective burn prevention program should include broad initiatives to raise awareness, identify risk factors, and develop and implement public health policies^(30)^. One study found that after caregivers participated in the Child Burn Prevention Program, the total number of burn-related risk factors in the home environment decreased by approximately 50%, while knowledge levels increased by 36.16%. This difference suggests that the program effectively reduces risk factors and enhances public knowledge and awareness^(31)^.

Based on the findings of this study, we recommend future research to investigate new biomarkers that could improve diagnostic accuracy in burn patients with suspected infection. We also encourage prospective studies to explore preventive interventions aimed at reducing sepsis incidence in this population.

### Study limitations

A limitation of this study is that we conducted it at a single center, reflecting a local experience and limiting its external validity. Additionally, the small sample size may have hindered the detection of minor differences not anticipated in the sample size calculation between the studied groups. However, the study’s strength lies in its methodological rigor and the daily data collection performed by a trained and qualified research team.

### Contributions to the field of nursing, health, or public health

Burn injuries significantly altered the SOFA score in these patients. The new Sepsis-3 criteria, which define sepsis as an increase of 2 or more points in the SOFA score, did not demonstrate sufficient discriminatory power for diagnosing sepsis or predicting mortality. Therefore, all infections in burn patients should be considered severe and managed as sepsis, regardless of SOFA score variation.

Caring for burn patients in emergency settings is complex. It requires specialized training for the nursing team, particularly for nurses, who are responsible for identifying patient needs, developing a care plan, supervising its implementation, and evaluating its effectiveness. The findings of this study can contribute not only to nursing practice but also to the development of care protocols in burn units. Standardizing the use of scoring systems at patient admission enables the healthcare team to provide individualized and necessary attention to each patient.

## CONCLUSIONS

Burn injuries significantly altered the SOFA score in the studied patients at hospital admission. The SIRS, qSOFA, and SOFA scores did not effectively identify infection in burn patients. A 2-point variation in the SOFA score had low sensitivity for detecting infection and predicting a high risk of death at hospital discharge. Therefore, all infections in burn patients should be considered severe and managed as sepsis, regardless of SOFA score variation. The identified risk factors for mortality at hospital admission were the clinical variables age, TBSA burned, and SOFA score. Increases in age, TBSA burned, and SOFA score were significantly associated with a higher risk of death.

## Data Availability

https://doi.org/10.48331/scielodata.NDLEMP
