# Supplementary material for: Analysis of the SOFA score, quick-SOFA, and SIRS criteria in burn patients with infection
Source: Rev Bras Enferm. 2025 Aug 8;78(3):e20240111. doi: 10.1590/0034-7167-2024-0111 (PMC12334202; doi:10.1590/0034-7167-2024-0111)
Supplement: Translation

# Translation: Análise do escore SOFA, quick-SOFA e critérios SIRS em pacientes queimados e com infecção

**DOI:** 10.1590/0034-7167-2024-0111pt

**Authors:** Francielli Mary Pereira Gimenez, Lucienne Tibery Queiroz Cardoso, Gilselena Kerbauy, Tiemi Matsuo, Cintia Magalhães Carvalho Grion

**Affiliations:** I Universidade Estadual de Londrina. Londrina, Paraná, Brasil

**Keywords:** Unidades de Queimados, Sepse, Triagem, Escores de Disfunção Orgânica, Síndrome de Resposta Inflamatória Sistêmica.

## Abstract

**Objetivos:**

avaliar o escore SOFA, qSOFA, critérios SIRS e fatores de risco para mortalidade hospitalar em vítimas de queimadura com suspeita de infecção internadas em unidade de terapia intensiva.

**Métodos:**

estudo longitudinal retrospectivo realizado em hospital público entre janeiro de 2017 e janeiro de 2020. Os escores para diagnóstico de sepse foram analisados em dois momentos: na admissão hospitalar e data da infecção.

**Resultados:**

dos 279 pacientes analisados, 251 tiveram infecção. Entre os pacientes com infecção, 145 pacientes apresentaram SIRS positivo na queimadura, permanecendo 112 positivos na primeira infecção. A variação do escore SOFA foi positiva em 187 pacientes na queimadura, e 34 permaneceram positivos na infecção.

**Conclusões:**

os escores nas datas da queimadura e infecção não mostraram variação de SIRS e SOFA compatível com o diagnóstico de sepse. Idade, superfície corporal queimada e SOFA foram fatores de risco independentes para mortalidade.

## INTRODUÇÃO

A pele possui função protetora contra os agentes externos, dentre eles os microrganismos e as agressões decorrentes do ambiente. Quando ocorre uma queimadura e consequente destruição da pele, o paciente torna-se mais susceptível a adquirir infecções, sendo a mais frequente e grave complicação em pacientes vítimas de queimaduras^(1)^.

A lesão por queimadura desencadeia resposta inflamatória exuberante e altera os marcadores inflamatórios clínicos e laboratoriais, mimetizando o diagnóstico de sepse^(2)^. Na ocorrência de infecção após uma lesão por queimadura, a identificação da sepse pode ser dificultada, pondo em dúvida o desempenho das ferramentas recomendadas para triagem e diagnóstico de sepse nesses pacientes.

A infecção com alto risco de morte é considerada sepse. O terceiro consenso internacional para definições de sepse e choque séptico, conhecido como Sepsis-3, define a sepse como disfunção orgânica potencialmente fatal causada por uma resposta orgânica desregulada a uma infecção. O diagnóstico de sepse é feito na presença de um foco suspeito ou confirmado de infecção associado a disfunção orgânica identificada como uma variação de dois ou mais pontos no escore *Sequential Organ Failure Assessment* (SOFA)^(3)^. Atualmente as ferramentas mais aplicadas para a triagem e diagnóstico de sepse no ambiente hospitalar são a *Systemic Inflammatory Response Syndrome* (SIRS), SOFA e quickSOFA (qSOFA), porém esses critérios não são específicos para os pacientes vítimas de queimaduras.

Criada na primeira definição de consenso de sepse, a SIRS é estabelecida como uma resposta inflamatória a uma variedade de insultos clínicos graves^(4)^, sendo essencial na triagem de pacientes potencialmente infectados.

O escore SOFA foi originalmente criado para avaliar disfunções de seis sistemas orgânicos em pacientes com suspeita de sepse. Já o qSOFA foi desenvolvido de forma simplificada com base no escore SOFA para ser aplicado especialmente nos setores de emergência a fim de identificar os pacientes com maiores chances de internação prolongada, maior risco de morte e necessidade de encaminhamento para cuidados intensivos^(3,5)^.

Os custos associados ao tratamento do paciente queimado são elevados, estimados em aproximadamente US$ 30 mil^(6)^. Mundialmente, a incidência de sepse no paciente grande queimado está entre 3% e 30%, sendo a pneumonia o foco infeccioso mais comum^(7)^. No Brasil, a sepse é a principal causa de morte nesses casos^(8)^, e o custo médio operacional mensal da unidade é de R$ 1.277.582,21^(9)^.

Diante desses fatos, é necessário analisar as ferramentas comumente aplicadas na identificação de infecção e sepse entre vítimas de queimadura hospitalizadas. O tratamento oportuno pode reduzir o risco de mortalidade, embora o reconhecimento precoce ainda seja um desafio.

## OBJETIVOS

Avaliar o escore SOFA, qSOFA, critérios SIRS e fatores de risco para mortalidade hospitalar em vítimas de queimadura com suspeita de infecção internadas em unidade de terapia intensiva.

## MÉTODOS

### Aspectos éticos

Esta pesquisa foi aprovada pela Comissão Nacional de Ética em Pesquisa (CONEP) e pelo comitê de ética da instituição por meio do Certificado de Apresentação para Apreciação Ética. Foi solicitada e aprovada a dispensa do Termo de Consentimento Livre e Esclarecido.

### Desenho, período e local do estudo

Estudo longitudinal retrospectivo, conduzido em uma unidade de terapia intensiva de queimados (UTIQ), entre janeiro de 2017 e janeiro de 2020. Foi utilizado o checklist STROBE da rede EQUATOR.

### População ou amostra; critérios de inclusão e exclusão

O hospital universitário da Universidade Estadual de Londrina (HU-UEL) é público, terciário e de grande porte, com 330 leitos. O centro de tratamento de queimados está inserido dentro do HU UEL, sendo uma unidade de referência no atendimento de pacientes queimados. É constituído de dez leitos de enfermaria, uma sala de pronto atendimento, uma sala de cirurgia e seis leitos de UTIQ. O estudo foi realizado na UTIQ. O referido hospital é classificado como de alta complexidade, centro de referência para o Sistema Único de Saúde (SUS) no norte do estado do Paraná, Brasil. Atende pacientes de cerca de 250 municípios do Paraná e mais de 100 cidades de outros estados. O centro de tratamento de queimados presta atendimento especializado a adultos e crianças vítimas de queimaduras de primeiro a terceiro grau.

A amostra constituiu-se dos prontuários de todos os pacientes vítimas de queimadura admitidos na UTIQ entre janeiro de 2017 e janeiro de 2020, totalizando 299 pacientes. Foram excluídos 20 pacientes, sendo 14 pelo critério de idade menor que 18 anos e 6 pacientes por dados incompletos no prontuário. Foi considerado prontuário incompleto aquele com dados faltantes que impossibilitaram o cálculo dos escores ou que não continha variáveis de interesse do estudo.

### Protocolo do estudo

A equipe de quatro pesquisadores que coletou os dados foi capacitada nas definições do estudo e no cálculo dos escores utilizados. Foi composta por um monitor, com função de auditoria do preenchimento e da qualidade dos dados; e por três colaboradores, que revezavam na coleta diária dos dados. O pesquisador auditor é enfermeiro e doutor em ciências da saúde, e os três pesquisadores colaboradores são alunos de graduação no curso de Medicina. Os dados demográficos e clínicos coletados foram: data da queimadura, data da internação hospitalar e da UTIQ, idade, sexo, peso e altura, *Abbreviated Burn Severity Index* (ABSI)^(10)^, Superfície Corpórea Queimada (SCQ), etiologia, agente e motivo da queimadura, além de presença de doença crônica.

Considerou-se “admissão hospitalar” a data de avaliação do evento da queimadura. Os escores SOFA, qSOFA e SIRS foram coletados em dois momentos: na data do evento da queimadura e na data do primeiro episódio de suspeita de infecção. Foram coletados data e foco das infecções bem como resultados laboratoriais de lactato e proteína C reativa (PCR). A data da alta hospitalar e o desfecho hospitalar foram anotados.

Os escores foram calculados de acordo com a descrição original dos autores^(3,4)^. Para o escore SOFA, foram coletados a razão entre a pressão arterial de oxigênio e a fração inspirada de oxigênio (PaO2 /FiO2) e uso de ventilação mecânica, contagem de plaquetas no sangue, dosagem sanguínea de bilirrubina total, nível de pressão arterial e uso de drogas vasoativas, escala de coma de Glasgow, dosagem sanguínea de creatinina e débito urinário. Para o escore qSOFA, foram coletadas a frequência respiratória, pressão arterial sistólica e alterações do estado de consciência. Já para SIRS, foram coletadas temperatura corporal, frequência cardíaca, frequência respiratória, contagem de leucócitos e presença de formas jovens de leucócitos no sangue. Os valores de ABSI, SOFA, qSOFA, SIRS e IMC foram calculados, e os resultados de lactato e PCR foram anotados conforme constavam no prontuário do paciente.

O escore SOFA basal foi considerado zero quando o paciente não apresentava comorbidade prévia que pontuasse nesse escore. A variação de 2 pontos ou mais foi considerada como ∆SOFA positivo, e a variação menor de 2 pontos foi tida como ∆SOFA negativo^(3)^. O ∆SOFA da queimadura foi avaliado na admissão da unidade pelo escore SOFA do dia da admissão em relação ao escore basal. O ∆SOFA para o diagnóstico de infecção foi avaliado pelo escore SOFA do dia da infecção em relação àquele de admissão na unidade.

Para cada item do escore qSOFA foi atribuído o valor de 1 ponto de acordo com os seguintes critérios: Frequência respiratória ≥ 22 movimentos/min; Pressão arterial sistólica ≤ 100 mmHg; e Alteração do estado de consciência. Foi considerado qSOFA positivo (+) se o escore apresentasse 2 ou 3 pontos^(3)^.

A SIRS foi pontuada considerando os seguintes critérios: Temperatura > 38 °C ou < 36 °C; Frequência cardíaca > 90 batimentos/min; Frequência respiratória > 20 movimentos/min ou PaCO_2_ < 32 mmHg (< 4,3 kPa); Leucócitos > 12.000 células/mm^3^ ou < 4.000 células/ mm^3^ ou > 10% de formas jovens. Foi considerada SIRS positiva (+) se o paciente apresentasse 2 ou mais pontos^(4)^.

A ocorrência de infecção foi o principal desfecho do estudo, sendo que foi considerada a primeira infecção ocorrida após a queimadura para as análises e comparações. O médico responsável pelo cuidado do paciente fez o diagnóstico de infecção com base na presença dos sintomas do paciente e nos critérios diagnósticos adotados pela comissão de controle de infecção hospitalar local. Como desfecho secundário, foi avaliada a condição vital na saída do hospital.

A definição de “comorbidade” deu-se pelos critérios do Índice de Comorbidade de Charlson (ICC)^(11,12)^. Por fim, o Índice de Massa Corporal foi a medida utilizada para identificar a obesidade, seus graus e as condições de sobrepeso.

### Análise dos resultados e estatística

Para o cálculo de tamanho de amostra, considerou-se um nível de significância bilateral de 95%, uma probabilidade de detecção de 80%, a razão entre os pacientes com ∆SOFA positivo e negativo de 1, a proporção de pacientes com ∆SOFA negativo e com infecção de 70% e a proporção de pacientes com ∆SOFA positivo e com infecção de 99,9%. O resultado foi uma amostra de 62 pacientes com infecção. Haja vista que a frequência da infecção em um CTQ pode variar de 20% a 65% dos pacientes admitidos, foi planejada uma amostra total de 250 pacientes admitidos na UTIQ para assegurar a observação de no mínimo 62 episódios de infecção.

Na estatística descritiva, as variáveis contínuas foram descritas pela média, desvio-padrão (DP), mediana e desvio interquartil (DIQ). Já as variáveis categóricas nominais foram descritas em frequência absoluta e relativa (%). Todas as variáveis foram apresentadas em tabelas.

Na estatística analítica, as variáveis categóricas foram comparadas utilizando o teste exato de Fisher. O teste não paramétrico (Mann-Whitney) foi aplicado para dados com distribuição não normal e/ou heterogeneidade de variâncias. O teste não paramétrico de Wilcoxon foi usado para amostras pareadas, a fim de avaliar as diferenças individuais. As variáveis categóricas foram analisadas com o teste de qui-quadrado. O teste de McNemar foi empregado para avaliar as discordâncias. Visando avaliar os fatores de risco para o desfecho “morte na saída do hospital”, realizou-se análise de riscos proporcionais pelo modelo de Cox, usando o método *stepwise* para seleção de variáveis. As variáveis que entraram no modelo foram idade, sexo feminino, superfície corporal queimada, presença de comorbidades e os escores SIRS, qSOFA e SOFA na admissão hospitalar.

## RESULTADOS

Durante o período do estudo, 299 pacientes foram admitidos na UTIQ e 20 pacientes excluídos, sendo analisados 279 pacientes entre janeiro de 2017 e janeiro de 2020. Entre os pacientes estudados, 182 (65,2%) sobreviventes tiveram alta hospitalar e 97 (34,8%) saíram devido a óbito. Dos 279 pacientes analisados no estudo, 186 (66,7%) eram do sexo masculino. A média de idade de toda a amostra foi de 43,3 anos (DP = 17,1). A média do IMC foi de 25,8 kg/m^2^ (DP = 4,4). As doenças crônicas mais frequentes foram tabagismo (58; 20,8%), etilismo (54; 19,4%), drogadição (43; 15,4%) e hipertensão arterial sistêmica (38; 13,6%) (Tabela 1).

**Tabela 1:** Média e desvio-padrão da idade e IMC do total de pacientes, dos sobreviventes e dos óbitos. Número e porcentagem segundo sexo e doenças crônicas do total de pacientes, dos sobreviventes e dos óbitos, Londrina, Paraná, Brasil, janeiro de 2017 a janeiro de 2020

Características	Total (n = 279)	Sobreviventes (n = 182)	Óbitos (n = 97)	Valor de *p*
Idade (Média ± DP)	43,3 ± 17,1	41,0 ± 15,7	47,8 ± 18,7	0,004**^a^**
Sexo n (%) Feminino Masculino	93 (33,3) 186 (66,7)	62 (66,7) 120 (64,5)	31 (33,3) 66 (35,5)	0,722^b^
IMC (Média ± DP)	25,8 ± 4,4	25,7 ± 4,6	25,9 ± 4,0	0,506^a^
Doenças crônicas n (%) Asma/DPOC Câncer Depressão Diabete *Mellitus* Drogadição Epilepsia Esquizofrenia Etilismo HAS HIV ICC Tabagismo	4 (1,4) 1 (0,4) 6 (2,2) 4 (1,4) 43 (15,4) 7 (2,5) 3 (1,1) 54 (19,4) 38 (13,6) 1 (0,4) 7 (2,5) 58 (20,8)	4 (2,2) - 4 (2,2) 3 (1,6) 25 (13,7) 6 (3,3) 2 (1,1) 30 (16,5) 25 (13,7) - 5 (2,7) 37 (20,3)	- 1 (1,0) 2 (2,1) 1 (1,0) 18 (18,6) 1 (1,0) 1 (1,0) 24 (24,7) 13 (13,4) 1 (1,0) 2 (2,1) 21 (21,6)	0,302^c^ 0,348^c^ 1,000^c^ 1,000^c^ 0,288^c^ 0,428^c^ 1,000^c^ 0,096^c^ 0,938^c^ 0,348^c^ 1,000^c^ 0,796^c^

DP - desvio-padrão; IMC - Índice de Massa Corporal; DPOC - doença pulmonar obstrutiva crônica; HAS - hipertensão arterial sistêmica; HIV - Human Immunodeficiency Virus; ICC - insuficiência cardíaca congestiva;

a Mann-Whitney;

b qui-quadrado;

c exato de Fisher.

No tocante ao tempo transcorrido entre o trauma da queimadura até a admissão na UTIQ, a média foi de 2,7 dias (DP = 3,5), e a média do escore ABSI foi de 6,7 (DP = 2,2). A média da SCQ foi de 25,6% (DP = 17,6). Quanto à lesão por inalação, 55 (19,7%) pacientes apresentaram essa lesão. Sobre a etiologia da queimadura, 83,1% foram por etiologia térmica. Os agentes da queimadura mais frequentes foram álcool (104; 37,5%), chama (45; 16,2%), gasolina (33; 11,9%) e líquido quente (32; 11,6%). Em relação ao motivo da queimadura, os acidentes domésticos foram os mais frequentes (58,5%), seguidos dos acidentes de trabalho (25,6%).

Dos 279 pacientes analisados, 251 (89,9%) foram diagnosticados com um ou mais episódios de infecção durante a internação. Não foi detectada presença de infecção nos pacientes do estudo na admissão hospitalar. A média do tempo entre a queimadura e a primeira infecção foi de 4,8 dias (DP = 4,0). O foco de infecção foi indeterminado em 39 (16%) casos. O foco pulmonar (107; 42%) foi o mais frequente, seguido da infecção de pele e partes moles (45; 18%), corrente sanguínea (32; 13%), trato urinário (26; 10%) e ouvido (02; 1%).

No que tange à variação dos exames laboratoriais, houve queda no valor médio do lactato e aumento no valor médio da PCR entre a queimadura e a primeira infecção documentada (Tabela 2).

**Tabela 2:** Mediana e desvio interquartil do lactato e da proteína C reativa na admissão e na 1ª infecção, Londrina, Paraná, Brasil, janeiro de 2017 a janeiro de 2020

Variáveis	n	Admissão	1ª Infecção	DIF	Valor de *p*
Lactato (Mediana ± DIQ)	250	2,0 ± 1,3	1,9 ± 1,1	-0,1 ± 0,8	0,012^a^
PCR (Mediana ± DIQ)	248	112,5 ± 195,8	197,9 ± 44,8	-14,8 ± (-102,5)	<0,001^a^

*DIF - Differential Items Functioning; DIQ - desvio interquartile; PCR - proteína C reativa; aWilcoxon.*

Na queimadura, a SIRS foi positiva em 153 (54,8%) pacientes e negativa em 126 (45,2%). Já na primeira infecção documentada, a SIRS positiva em 170 (67,7%) pacientes e negativa em 81 (32,3%). No evento da queimadura, o escore qSOFA foi negativo em 172 (68,5%) pacientes e positivo em 79 (31,5%) pacientes. Já na primeira infecção documentada, o qSOFA foi negativo em 147 (58,6%) pacientes e positivo em 104 (41,4%) pacientes. Quanto ao ∆SOFA no evento da queimadura, 64 (25,5%) pacientes tiveram uma variação no escore menor que 2 pontos (∆SOFA negativo) e 187 (74,5%) pacientes apresentaram variação na pontuação maior ou igual a 2 (∆SOFA positivo). Na primeira infecção documentada, o ∆SOFA foi negativo em 192 (76,5%) pacientes e positivo em 59 (23,5%) (Tabela 3).

**Tabela 3:** Número e porcentagem do SIRS, qSOFA e ∆SOFA na admissão e na 1ª infecção, Londrina, Paraná, Brasil, janeiro de 2017 a janeiro de 2020

Variáveis	Admissão	1ª Infecção
SIRS, n (%)		
Positivo	153 (54,8)	170 (67,7)
Negativo	126 (45,2)	81 (32,3)
qSOFA, n (%)		
Positivo	172 (68,5)	147 (58,6)
Negativo	79 (31,5)	104 (41,4)
∆SOFA, n (%)		
< 2	64 (25,5)	192 (76,5)
≥ 2	187 (74,5)	59 (23,5)

*SIRS - Systemic Inflammatory Response Syndrome; qSOFA - quick Sequential Organ Failure Assessment; SOFA - Sequential Organ Failure Assessment.*

*∆SOFA da admissão = SOFA admissão - SOFA basal. ∆SOFA da infecção = SOFA infecção - SOFA admissão.*

Na análise dos escores, 145 (57,7%) pacientes apresentaram SIRS positivo na queimadura, ao passo que 112 (44,6%) permaneceram positivo e 33 (13,1%) passaram a apresentar SIRS negativo na primeira infecção documentada. Com o qSOFA, 79 (31,5%) pacientes tiveram qSOFA positivo na queimadura, dos quais 62 (24,7%) permaneceram positivos e 17 (6,8%) passaram a apresentar qSOFA negativo na primeira infecção documentada. Para o escore SOFA, 187 (74,5%) pacientes apresentaram ∆SOFA positivo na queimadura, dos quais 34 (13,5%) permaneceram positivos e 153 (61,0%) passaram a ter ∆SOFA negativo na primeira infecção documentada (Tabela 4).

**Tabela 4:** Número e porcentagem das variáveis SIRS, qSOFA e ∆SOFA positivas na admissão e no diagnóstico de infecção, Londrina, Paraná, Brasil, janeiro de 2017 a janeiro de 2020

Admissão	Infecção		Valor de *p*
	Variável positiva	Variável negativa	
SIRS Positiva Negativa	112 (44,6%) 58 (23,1%)	33 (13,1%) 48 (19,1%)	0,011^a^
qSOFA Positivo Negativo	62 (24,7%) 42 (16,7%)	17 (6,8%) 130 (51,8%)	0,002^a^
∆SOFA ≥ 2 Positivo Negativo	34 (13,5%) 25 (10,0%)	153 (61,0%) 39 (15,5%)	<0,001^a^

*SIRS - Systemic Inflammatory Response Syndrome; qSOFA - quick Sequential Organ Failure Assessment; SOFA - Sequential Organ Failure Assessment.*

∆SOFA da admissão = SOFA admissão - SOFA basal. ∆SOFA da infecção = SOFA infecção - SOFA admissão.

a McNemar.

Observou-se maior taxa de mortalidade entre os pacientes com infecção e ∆SOFA positivo (49,2%) comparados aos pacientes com infecção e ∆SOFA negativo (34,9%; p = 0,049). A taxa de mortalidade dos pacientes com infecção foi elevada nos dois grupos, porém foi maior entre os pacientes com uma variação do SOFA de 2 pontos ou mais.

Para analisar os fatores de risco para morte foi realizada regressão logística usando o método *stepwise*. Os fatores independentes associados a morte no desfecho hospitalar mostram que cada ano adicional de idade aumentou 2,6% na mortalidade, cada um por cento de aumento de SCQ aumentou em 3,6% as chances de morte e cada ponto adicional no escore SOFA aumentou a mortalidade em 12,7% (p < 0,001) (Tabela 5).

**Tabela 5:** *Hazard ratio* **e intervalo de confiança em função dos fatores de risco para morte, no modelo completo e no modelo** *stepwise* **, Londrina, Paraná, Brasil, janeiro de 2017 a janeiro de 2020**

Variáveis	Modelo Completo Hazard ratio (95% IC)	Valor de *p*	Modelo Stepwise Hazard ratio (95% IC)	Valor de *p*
Idade	1,021 (1,008 - 1,034)	<0,001	1,026 (1,014 - 1,039)	< 0,001
Sexo feminino	0,800 (0,511 - 1,253)	0,330		
SCQ	1,029 (1,018 - 1,040)	<0,001	1,036 (1,025 - 1,047)	< 0,001
Comorbidade	1,141 (0,690 - 1,886)	0,607		
SIRS admissão	0,579 (0,334 - 1,003)	0,051	0,574 (0,336 - 0,981)	0,043
qSOFA admissão	0,917 (0,636 - 1,320)	0,640		
SOFA admissão	1,107 (1,026 - 1,195)	0,009	1,127 (1,067 - 1,190)	< 0,001

*IC - intervalo de confiança; SCQ - superfície corporal queimada; SIRS - Systemic Inflammatory Response Syndrome; qSOFA - quick Sequential Organ Failure Assessment; SOFA - Sequential Organ Failure Assessment.*

Entre os exames laboratoriais e escores prognósticos avaliados, foi calculada a área sob a curva ROC, porém todas as curvas apresentaram baixa acurácia para predizer infecção ou morte. A área sob a curva ROC do escore SOFA da queimadura para predizer infecção foi de 0,323, do lactato na queimadura para predizer infecção foi de 0,371 e da PCR da queimadura para predizer infecção foi de 0,330. A área sob a curva ROC do escore SOFA da queimadura para predizer morte foi de 0,252, do lactato da queimadura para predizer morte foi de 0,337 e da PCR da queimadura para predizer morte foi de 0,396.

## DISCUSSÃO

Os resultados deste estudo mostram que mais da metade dos pacientes já apresentavam os marcadores inflamatórios e de disfunção orgânica alterados logo após o evento da queimadura, mesmo sem a presença de infecção. No momento do diagnóstico da infecção, esses marcadores permaneceram positivos em alguns pacientes, mas se tornaram negativos em outros. Nos pacientes com marcadores negativos no evento da queimadura, nem todos se tornaram positivos para a identificação de sepse no momento da infecção. Por outro lado, os pacientes queimados com infecção tiveram alto risco de morte, independentemente da positividade dos marcadores analisados. Esses dados sugerem que as ferramentas usuais de triagem e diagnóstico de sepse apresentam baixo desempenho nos pacientes vítimas de queimadura com suspeita de infecção. Idade, superfície corporal queimada e SOFA foram fatores de risco independentes para mortalidade hospitalar.

A variação do SOFA de 2 ou mais pontos apresentou frequência semelhante nos eventos da queimadura e da infecção entre os pacientes estudados, sendo este o critério adotado para o diagnóstico de sepse na reunião de consenso Sepsis-3^(3)^. Outro estudo tampouco detectou bom desempenho do escore SOFA maior ou igual a 2 pontos para identificação de sepse em pacientes queimados^(13)^. Os autores sugerem que o escore SOFA maior ou igual a 6 possa ser um melhor critério para o diagnóstico de sepse nesses pacientes. Nossos dados indicam que pacientes queimados com infecção e variação do escore SOFA menor que 2 podem apresentar alto risco de morte, ou seja, isso se dá mesmo sem preencherem os critérios de diagnóstico de sepse pelas definições do consenso Sepsis-3. Dessa forma, não seria adequado aumentar a variação do escore SOFA para 6 pontos, o que pioraria a sensibilidade do diagnóstico de sepse em nossos pacientes.

O escore SOFA da queimadura permaneceu como fator independente associado a morte, portanto pode ser considerado um critério para avaliação desses pacientes. Esse escore demonstra maior acurácia prognóstica para mortalidade intra-hospitalar quando comparado aos critérios SIRS e qSOFA em pacientes admitidos em uma unidade de terapia intensiva^(14)^. Além do escore SOFA, idade e superfície corpórea queimada foram fatores associados ao óbito nos pacientes queimados do nosso estudo, em concordância com outros autores^(5)^. A idade avançada e a destruição da barreira natural do organismo aumentam os riscos para infecções e outras complicações, elevando assim a taxa de morbidade e mortalidade no paciente queimado^(15)^.

Neste estudo, os casos de queimadura foram mais frequentes em homens. Tal resultado corrobora os encontrados na literatura nacional, já que estudos epidemiológicos sobre o perfil das vítimas de queimaduras vêm mostrando uma maior incidência desses acidentes no sexo masculino^(16,17)^. A média da idade dos pacientes acometidos é semelhante aos achados de outros estudos, com prevalência na população adulta^(18,19)^. Tal situação reflete negativamente nos aspectos socioeconômico e familiar, uma vez que essa faixa etária populacional está ativa no mercado de trabalho e/ou nos afazeres do lar, respondendo pela geração da renda familiar e cuidado da família^(20)^. Quanto às doenças crônicas, nota-se que o tabagismo, etilismo e drogadição foram as mais prevalentes. Um estudo mostrou que 19,37% dos pacientes queimados apresentavam hábito tabagista; 15,03%, hábito etilista; e 6,83%_,_ consumo de drogas^(16)^.

No presente estudo, a média da SCQ foi superior a 20%, semelhante a resultados encontrados por outros autores^(18)^. Quanto à etiologia, a térmica foi a mais prevalente, e os acidentes domésticos foram os mais frequentes. Esses resultados também foram descritos por outros autores^(19)^. A média do escore “prognóstico de queimaduras ABSI” foi semelhante à pontuação encontrada em outros estudos^(18,20)^. A média desse escore enquadra esses pacientes dentro da classificação “ameaça à vida moderadamente severa”, necessitando de vigilância contínua e cuidado especializado.

Complicações respiratórias constituem uma realidade nos pacientes queimados, principalmente quando ocorre inalação de fumaça. Porém, pacientes grandes queimados sem inalação de fumaça frequentemente apresentam complicações pulmonares devido à imobilidade e hipoventilação causada pela dor. A necessidade de vários procedimentos anestésicos pode levar à atelectasia e consequente pneumonia. O emprego de sedativos e de bloqueadores neuromusculares propicia a retenção de secreções brônquicas e aspiração traqueal^(21)^. Outros autores também descreveram o pulmão como o sítio de infecção mais frequente^(13,22)^. O tempo médio de internamento, quando comparado com a média de superfície corpórea queimada, foi compatível com os resultados encontrados por outros autores^(18)^.

A mediana do lactato no momento do diagnóstico de infecção apresentou uma redução pequena quando comparada com a mediana da queimadura, enquanto a mediana da PCR na infecção foi superior em relação a mediana da queimadura. Com a lesão térmica, ocorre o recrutamento de neutrófilos e de macrófagos. Essas células liberam citocinas inflamatórias, que ativam a produção de PCR, e a inflamação leva à hipoperfusão tecidual com formação de lactato^(2,23)^. Outros autores descrevem níveis elevados de PCR em pacientes queimados mesmo na ausência da infecção^(24-26)^. Sendo assim, PCR e lactato não devem ser utilizados como única ferramenta diagnóstica para investigar presença de infecção em pacientes queimados^(24,25)^. A baixa acurácia das curvas ROC do escore SOFA, lactato e PCR para predizer infecção ou morte sugere a necessidade de buscar novos indicadores.

O diagnóstico de complicações infecciosas e a identificação dos sinais de sepse são um grande desafio no cuidado do paciente grande queimado. Isoladamente cada variável não apresenta sensibilidade e especificidade para a detecção da sepse, mas, quando utilizadas de forma associada, é possível melhorar a acurácia do diagnóstico^(27)^. O uso de ferramentas de alerta precoce na prática clínica dos enfermeiros tem grande repercussão na tomada de decisão em momentos críticos, favorecendo o cuidado nos diversos ambientes intra-hospitalares; sendo assim, essa utilização é capaz de influenciar positivamente a assistência prestada ao paciente e o raciocínio clínico à beira do leito^(28)^.

Apesar dos avanços na prevenção, identificação precoce e tratamento da sepse, nota-se que ainda há grande incidência dessa condição no ambiente hospitalar, principalmente entre os pacientes queimados, que estão mais susceptíveis a essa complicação^(29)^. A padronização do uso de escores na admissão do paciente permite a criação de protocolos para que a equipe multidisciplinar planeje, oriente cuidados e dê atenção a cada paciente individualmente, agindo de forma ágil e qualitativa na utilização de recursos para aumentar a sobrevida dessa população^(18)^.

Vale ressaltar que as queimaduras são evitáveis e a prevenção pode reduzir a sua incidência. Um programa eficaz de prevenção de queimaduras deve incluir amplos esforços para melhorar a conscientização, identificar os fatores de risco e desenvolver e aplicar políticas públicas de saúde^(30)^. Um estudo observou que, ao aplicar o Programa de Prevenção de Queimaduras Infantis aos cuidadores, o número total de fatores de risco relacionados às causas de queimaduras no ambiente domiciliar diminuiu aproximadamente 50%, enquanto o nível de conhecimento aumentou 36,16%. Tal diferença sugere que o programa tem sucesso na redução dos fatores de risco e mostra-se eficaz para aumentar o conhecimento e a conscientização da população^(31)^.

Com base nos resultados do presente artigo, sugerimos estudos futuros para investigar novos biomarcadores que melhorem a precisão diagnóstica em pacientes queimados com suspeita de infecção. Também recomendamos estudos prospectivos para explorar intervenções preventivas que reduzam a incidência de sepse nessa população.

### Limitações do estudo

A limitação refere-se ao fato de ser um estudo de centro único, portanto reflete uma experiência local, limitando sua validade externa. Além disso, a amostra reduzida pode ter impedido a detecção de pequenas diferenças não previstas no cálculo amostral entre os grupos estudados. Entretanto, o ponto forte do estudo está no rigor metodológico e na coleta de dados diária por equipe treinada e capacitada para função.

### Contribuições para a área da enfermagem, saúde ou política pública

A queimadura alterou significativamente o escore SOFA desses pacientes. Os novos critérios propostos no Sepsis-3 de um aumento de 2 pontos ou mais no escore SOFA não apresentaram poder de discriminação para o diagnóstico de sepse e para o desfecho “morte”. Dessa forma, entende se que toda infecção no paciente queimado deve ser considerada grave, necessitando ser tratada como sepse independentemente da variação do escore SOFA.

A assistência ao paciente queimado no cenário da emergência é complexa e exige um preparo da equipe de enfermagem, principalmente do enfermeiro, que é responsável por elencar as demandas do paciente, criar o plano de cuidado, supervisionar sua execução e avaliar sua eficácia. Os resultados do presente estudo podem não apenas contribuir para a prática de enfermagem, mas também auxiliar no desenvolvimento de protocolos de atendimento em unidades de queimados, já que a padronização do uso de escores na admissão favorece para que a equipe dê a atenção necessária e individual a cada paciente.

## CONCLUSÕES

A queimadura alterou significativamente o escore SOFA dos pacientes estudados na admissão hospitalar. Os escores SIRS, qSOFA e SOFA não identificaram o evento de infecção no paciente queimado. A variação de 2 pontos no escore SOFA teve baixa sensibilidade para identificar pacientes com infecção e alto risco de morte na saída do hospital. Dessa forma, entende-se que toda infecção no paciente queimado deve ser considerada grave, necessitando ser tratada como sepse independentemente da variação do escore SOFA. Os fatores de risco para mortalidade identificados na admissão hospitalar foram as variáveis clínicas idade, SCQ e SOFA. Cada aumento na idade, na SCQ e na pontuação do escore SOFA foi significativamente associado a um risco maior de morte.

## Data Availability

https://doi.org/10.48331/scielodata.NDLEMP
